# Association Between Internet Gaming Disorder and Suicidal Ideation Mediated by Psychosocial Resources and Psychosocial Problems Among Adolescent Internet Gamers in China: Cross-Sectional Study

**DOI:** 10.2196/48439

**Published:** 2024-09-19

**Authors:** Yanqiu Yu, Anise M S Wu, Vivian W I Fong, Jianxin Zhang, Ji-bin Li, Joseph T F Lau

**Affiliations:** 1 Department of Preventive Medicine and Health Education School of Public Health Fudan University Shanghai China; 2 Department of Psychology Faculty of Social Sciences University of Macau Macao China; 3 Centre for Cognitive and Brain Sciences Institute of Collaborative Innovation University of Macau Macao China; 4 Center for Health Behaviours Research Jockey Club School of Public Health and Primary Care The Chinese University of Hong Kong Hong Kong China; 5 West China School of Public Health Sichuan University Chengdu China; 6 State Key Laboratory of Oncology in South China, Collaborative Innovation Center for Cancer Medicine Department of Clinical Research Sun Yat-sen University Cancer Center Guangzhou China; 7 Public Mental Health Center School of Mental Health Wenzhou Medical University Wenzhou China; 8 Zhejiang Provincial Clinical Research Center for Mental Disorders The Affiliated Wenzhou Kangning Hospital Wenzhou Medical University Wenzhou China

**Keywords:** internet gaming disorder, suicidal ideation, adolescents, mediation, structural equation modelling, resilience, loneliness, social support, social anxiety

## Abstract

**Background:**

Adolescent internet gaming disorder (IGD) was associated with severe harm, including suicidal ideation. While suicidal ideation was predictive of completed suicides, further research is required to clarify the association between IGD and suicidal ideation among adolescents, as well as the mechanisms involved.

**Objective:**

This study aimed to investigate the understudied association between IGD and suicidal ideation, as well as novel mechanisms associated with it, among Chinese adolescent internet gamers through psychosocial coping resources and psychosocial problems.

**Methods:**

An anonymous, self-administered, cross-sectional survey was conducted among secondary school students who had played internet games in the past year in Guangzhou and Chengdu, China (from October 2019 to January 2020). In total, 1693 adolescent internet gamers were included in this study; the mean age was 13.48 (SD 0.80) years, and 60% (n=1016) were males. IGD was assessed by the 9-item Internet Gaming Disorder Checklist of the *DSM-5* (*Diagnostic and Statistical Manual of Mental Disorders* [Fifth Edition]), while a single item assessed suicidal ideation: “Have you ever considered committing suicide in the past 12 months?” Univariate and multivariate logistic regression associations were conducted to test the significance and directions of the potential factors for suicidal ideation. The mediation mechanism was examined by structural equation modeling.

**Results:**

Among all participants, the prevalence of IGD and suicidal ideation was 16.95% (287/1693) and 43.06% (729/1693), respectively. IGD cases were 2.42 times more likely than non-IGD cases to report suicidal ideation (adjusted odds ratio [OR] 2.42, 95% CI 1.73-3.37). Other significant factors of suicidal ideation included psychosocial coping resources (resilience and social support, both adjusted OR 0.97, 95% CI 0.96-0.98) and psychosocial problems (social anxiety: adjusted OR 1.07, 95% CI 1.05-1.09; loneliness, adjusted OR 1.13, 95% CI 1.10-1.16). The association between IGD and suicidal ideation was partially mediated by 3 indirect paths, including (1) the 2-step path that IGD reduced psychosocial coping resources, which in turn increased suicidal ideation; (2) the 2-step path that IGD increased psychosocial problems, which in turn increased suicidal ideation; and (3) the 3-step path that IGD reduced psychosocial coping resources which then increased psychosocial problems, which in turn increased suicidal ideation, with effect sizes of 10.7% (indirect effect/total effect: 0.016/0.15), 30.0% (0.05/0.15), and 13.3% (0.02/0.15), respectively. The direct path remained statistically significant.

**Conclusions:**

IGD and suicidal ideation were alarmingly prevalent. Evidently and importantly, IGD was a significant risk factor for suicidal ideation. The association was partially explained by psychosocial coping resources of resilience and social support and psychosocial problems of social anxiety and loneliness. Longitudinal studies are needed to confirm the findings. Pilot randomized controlled trials are recommended to evaluate the effectiveness of interventions in reducing suicidal ideation by reducing IGD, improving psychosocial coping resources, and reducing psychosocial problems investigated in this study.

## Introduction

Adolescent suicide is the fourth leading cause of death among adolescents aged 15-19 years [1]. A review including studies conducted in 15 countries reported age-standardized suicide rates among male and female youths aged 15-29 years ranging from 2.4 to 51 per 100,000 and from 1.1 to 16.4 per 100,000, respectively [2]. A national report showed the prevalence of suicide ranging from 8 to 11.8 per 100,000 among adolescents aged 15-20 years in China [3,4]. The spectrum of suicide includes conditions ranging from suicidal ideation (having thoughts of ending one’s own life), suicidal intent (having the specific intention of ending one’s own life, often including planning or preparation), suicidal attempt (having attempted to end one’s own life but not resulting in death), to death [5]. Suicidal ideation increases the risk of suicidal behaviors [6]. A better understanding of the risk factors of adolescent suicidal ideation and related mechanisms is warranted.

Addictive behaviors (eg, substance use and internet addiction) are well-documented risk factors for suicidal ideation [7,8]. Internet gaming disorder (IGD), which is a subtype of gaming disorder, has been included as a mental disorder in the *ICD-11* (*International Classification of Diseases* [*11th Revision*]) published by the World Health Organization in 2018 [9]. It was associated with harmful behavioral and psychological consequences, including suicidal ideation [10]. A review of 12 cross-sectional studies found moderate-to-strong positive associations between problematic gaming and suicidal ideation [11]. However, only 1 study targeted Chinese adolescents [12]. In addition, only 1 longitudinal study conducted among Swedish adults looked at such a relationship and found that behavioral addiction (including IGD) at age 25 years significantly predicted suicidal ideation at age 28 years [13]. Understanding the mechanisms between IGD and suicidal ideation would guide the development of effective interventions. To our knowledge, however, only 1 study conducted in China had investigated 1 such potential mechanism (ie, IGD was associated with increased insomnia symptoms that led to a higher risk of depression, which in turn elevated the risk of suicidal ideation) among adolescents [12]. Research is greatly warranted to fill this knowledge gap.

The psychosocial problems of social anxiety and loneliness are potential mediators between IGD and suicidal ideation. Loneliness refers to distressing feelings that arise when there is a lack of intimate or satisfactory social connections [14]. In contrast, social anxiety refers to the perceived fear of being watched and judged by others in social situations [15]. Loneliness increases the risk of social anxiety and vice versa [16]. Furthermore, they often come together and are positively associated with mental problems, including thoughts of self-harm and suicide [17-19]. In addition, there is further support for the above-proposed mediation. First, pathological internet use (including IGD) has been postulated to cause deficient face-to-face social interactions that would lead to social isolation [20,21]. Longitudinal studies have also reported that adolescent IGD predicted social anxiety and loneliness [22,23]. Second, according to the Interpersonal-Psychological Theory of Suicide, suicidal thoughts are determined by perceived burdensomeness to others or society and thwarted belongingness [24], which is potentially associated with social anxiety and loneliness [24,25].

It is plausible that psychosocial coping resources (eg, resilience and social support) will further mediate the association between IGD and psychosocial problems (eg, social anxiety and loneliness). In that case, a serial mediation between IGD and suicidal ideation, first by psychosocial coping resources and then by social anxiety and loneliness, would occur. Regarding psychosocial coping resources, resilience is a personal resource referring to the ability to withstand and bounce back from difficult life events [26]. Social support is an interpersonal resource referring to receiving assistance or comfort from interpersonal relationships within an individual’s social network [27,28]. A couple of theories support the proposed mediation between IGD and psychosocial problems through psychosocial coping resources. First, potential functional impairments of IGD (eg, problems in personal life, social relationships, and academic performance) can be seen as strong stressors [29]. According to the Resource Deterioration Model, the presence of such stressors (functional impairments of IGD in this case) would diminish coping resources (resilience and social support in this case) [30,31]. Second, the Conversation of Resource theory postulates that losses in personal and interpersonal resources (those resulting from IGD in this case) would cause psychosocial problems (social anxiety and loneliness in this case) and suicidal ideation [32-35]. Thus, this study contended that IGD would reduce both personal coping resources (resilience) and interpersonal coping resources (social support), which would then increase social anxiety and loneliness, which would, in turn, increase suicidal ideation.

The proposed mechanisms of this study have further empirical support. First, a study conducted in Taiwan reported that the IGD group showed a higher level of stress and a lower level of resilience than the non-IGD group [36], while a longitudinal study found that IGD predicted a lower level of social support among Chinese university students [37]. Second, resilience and social support were negatively associated with social anxiety [38,39], loneliness [40,41], and suicidal ideation [42,43]. Third, social anxiety and loneliness were significantly associated with suicidal ideation [17,44]. Furthermore, extant literature has reported significant mediations between psychosocial resources and mental disorders through psychosocial problems. For instance, loneliness significantly mediated the association between perceived social support and depression among Chinese rural-to-urban migrants [45].

Given the background, this study investigated the prevalence of IGD and suicidal ideation among adolescent internet gamers in 2 Chinese cities. Factors of suicidal ideation were investigated, including (1) IGD, (2) a total of 2 types of psychosocial coping resources (resilience and social support), and (3) a total of 2 types of psychosocial problems (social anxiety and loneliness). The tested mediation model contains 3 mediation paths (indirect effects) between IGD and suicidal ideation that are (1) a 2-step path, in which IGD would reduce psychosocial resources (resilience and social support), which would, in turn, increase suicidal ideation; (2) a 2-step path, where IGD would increase psychosocial problems (social anxiety and loneliness), which would, in turn, increase suicidal ideation; and (3) a 3-step serial mediation path postulating that IGD would reduce psychosocial resources (resilience and social support), which would then increase psychosocial problems (social anxiety and loneliness), which would, in turn, increase suicidal ideation. In addition, the direct path from IGD to suicidal ideation was tested.

## Methods

### Participants and Data Collection

A cross-sectional survey was conducted among junior middle school students in 2 metropolises (Guangzhou and Chengdu) in China from October 2019 to January 2020. Guangzhou and Chengdu had population sizes of 15.3 and 16.3 million in 2019, respectively. All eighth-grade students from 4 Guangzhou schools and all seventh to ninth grade students from 3 Chengdu schools were conveniently selected and invited to participate in this survey. Notably, the Guangzhou sample has been used in 2 previous publications whose topics were completely different from this study; one investigated the impacts of the medicalization of IGD [46], and the other validated an assessment tool for potential resource losses related to gaming time reduction [47]. The data collection procedure was, hence, the same as those in these published studies and was briefly introduced here.

In the absence of teachers, students self-administered an anonymous, structured questionnaire using paper and pencil in the classroom. Well-trained fieldworkers briefed the students about the anonymous and voluntary nature and logistics of the study, that there would be no consequences of refusing or quitting this survey at any time if wished, and that the return of the completed questionnaires would imply informed consent. The questionnaire took 30 to 40 minutes to complete. The fieldworkers also assisted with inquiries from the students if needed and did the quality check when the students submitted the questionnaire.

There were 3039 completed questionnaires (a response rate of 99.09%, 3039/3067), among which 74 participants (2.44%) were removed from data analysis due to missing data in ≥20% of the questionnaire items (n=60, 1.97%) or any missing data in the key variables (eg, IGD and suicidal ideation; n=14, 0.46%). Out of the 2965 valid responses, 1272 (42.90%) participants had not played internet games in the past 12 months (nongamers) and were excluded from data analysis. The effective sample size of this study was hence 1693 (Guangzhou: 55.46%, n=939; Chengdu: 44.54%, n=754).

### Ethical Considerations

This study involved informed consent from 3 parties. First, school consent was obtained from school principals before the investigation. Second, parents were informed about the survey, and the opt-out procedure was exercised. Third, students were briefed by the fieldworkers about the anonymous and voluntary nature of this study and that the submission of a completed questionnaire would indicate informed consent, such information was also printed on the cover page of the questionnaire; no written informed consent was obtained to avoid disclosure of personal identity. No incentive was provided to school coordinators, school staff, parents, and students. This project and the above-informed consent procedures were approved by the Survey and Behavioral Research Ethics Committee of the Chinese University of Hong Kong in 2019 (SBRE-18-430).

### Measurements

#### Sociodemographics

Information was collected about age (years), sex, whether living with both parents or a single-parent family, and self-rated household financial situations relative to the participant’s classmates (5 points: very poor, poor, moderate, good, or very good). Notably, the relative financial situation had much greater predictive validity for self-reported health and well-being than the absolute financial situation [48].

#### Suicidal Ideation

Furthermore, 1 item assessed, “Have you ever considered committing suicide in the past 12 months?” (yes or no were the response options). The single-item assessment of suicidal ideation has shown predictive validity for passive and active suicidal ideation as well as depression [49]. In addition, previous studies have used the same or similar single items to assess suicidal ideation [12,50,51].

#### IGD

It was assessed by the 9-item IGD Checklist of the *DSM-5* (*Diagnostic and Statistical Manual of Mental Disorders* [Fifth Edition]). IGD was defined as the endorsement of at least 5 of the 9 types of *DSM-5* symptoms (preoccupation, withdrawal, tolerance, inability to control internet gaming, prioritization over other activities, continuation of internet gaming despite adverse consequences, deception of internet gaming time, avoidance, and significant loss due to internet gaming) in the past 12 months (yes or no response options) [52]. The Chinese version of the checklist has been validated among Chinese adolescents and yielded satisfactory psychometric properties [53]. The Cronbach α of the scale was 0.79 in this study.

#### Resilience

It was assessed by the 10-item Connor-Davidson Resilience Scale [26]; its Chinese version has been validated among adolescents and showed satisfactory psychometric properties [54]. A sample item is “I can adapt to change.” The items were rated on a 5-point Likert scale (0=never to 4=always). The Cronbach α of the scale was 0.93 in this study.

#### Social Support

It was assessed by 2 subscales of the Multidimensional Scale of Perceived Social Support, which assessed perceived social support from family members and friends [27]. Its Chinese version has been validated among adolescents in China and showed acceptable psychometric properties [55]. A sample item is “My family members/friends really try to help me.” The items were rated on a 7-point Likert scale (1=extremely disagree to 7=extremely agree). The Cronbach α of the scale was 0.93 in this study.

#### Social Anxiety

It was assessed by using the 9-item social anxiety subscale of the Multidimensional Anxiety Scale for Children [56]; the Chinese version has been validated among adolescents in China and showed good psychometric properties [57]. A sample item is “I’m afraid other people will think I’m stupid.” The items were rated on a 4-point Likert scale (0=disagree to 3=always agree). The Cronbach α of the scale was 0.92 in this study.

#### Loneliness

It was assessed by using the 8-item short-form of the UCLA (University of California, Los Angeles) Loneliness Scale; the Chinese version has been validated among adolescents in China, which showed acceptable psychometric properties [58]. A sample item is “I feel isolation from others.” The items were rated on a 4-point Likert scale (0=never to 3=always); higher scores indicated higher levels of loneliness. The Cronbach α of the scale was 0.78 in this study.

#### Probable Moderate or Above Depression

It was assessed by the 9-item Patient Health Questionnaire (PHQ-9), which is a multipurpose instrument for screening, diagnosing, and monitoring the severity of depression. Its Chinese version has been validated in adolescents and showed good psychometric properties [59,60]. A sample item is “Little interest or pleasure in doing things.” The items were rated on a 4-point Likert scale on the frequency of having potential symptoms of depression in the past 2 weeks (0=not at all to 3=nearly every day). Probable moderate or above depression was defined as a PHQ-9 score ≥10 in this study; it has been used in several previous studies [61,62]. The Cronbach α of the scale was 0.93 in this study.

### Statistical Analysis

IGD status and probable moderate or above depression were used in binary forms. Mean (SD, range) and frequency (proportion) were used to statistically describe continuous and categorical variables, respectively. The Chi-square (*χ^2^*) test and independent-sample *t* test were performed to examine between-group differences regarding categorical and continuous variables, respectively. Pearson correlation coefficients (*r*) were generated to assess correlations between variables. Univariate and multivariate logistic regression analyses (adjusted for sociodemographics and probable moderate or above depression) were conducted to test the significant factors (IGD and psychosocial factors) for suicidal ideation. Unadjusted odds ratios (ORs) and adjusted ORs and their respective 95% CIs were presented.

Structural equation modeling (SEM) was fit to test the underlying mechanisms between IGD and suicidal ideation through the 4 psychosocial variables. The estimator of weighted least square mean and variance was used in SEM. A total of 2 latent variables were created: (1) the latent variable of psychosocial resources was generated from the scale scores of both resilience and social support, and (2) the latent variable of psychosocial problems was generated from the scale scores of both social anxiety and loneliness. The goodness-of-fit of the SEM model was evaluated by comparative fit index (CFI) ≥0.90 and root-mean-square error of approximation (RMSEA) ≤0.08 [63,64]. Standardized path coefficients (β) were reported.

SPSS Statistics (version 23.0; IBM Corp) and Mplus (version 7.0; Muthen & Muthen) were used for statistical analyses; *P*<.05 was considered statistically significant.

## Results

### Descriptive Statistics: Sociodemographics

Among all participants, the mean age was 13.48 (SD 0.80; range 10-19) years. Over half of them were male (60%, 1016/1693); about one-fifth did not live with both parents (20.08%, 340/1693) or lived in a single-parent family (15.95%, 270/1693); 12.52% (212/1693) considered their household financial situation poorer or much poorer relative to their classmates (Table 1).

**Table 1:** Descriptive statistics.

		Overall (N=1693), n (%)	IGD^a^			
			Yes (n=287, 16.95%), n (%)	No (n=1406, 83.05%), n (%)	*P* of *χ*^*2*^	
**Studied sites**						<.001
	Guangzhou	939 (55.46)	102 (35.54)	837 (59.53)		
	Chengdu	754 (44.54)	185 (64.46)	569 (40.47)		
**Sex**						<.001
	Female	668 (39.46)	80 (27.87)	588 (41.82)		
	Male	1016 (60.0)	203 (70.73)	813 (57.82)		
	Missing data	9 (0.54)	4 (1.39)	5 (0.36)		
**Living with both parents**						.001
	Yes	1342 (79.27)	205 (71.43)	1137 (80.87)		
	No	340 (20.08)	78 (27.18)	262 (18.63)		
	Missing data	11 (0.65)	4 (1.39)	7 (0.50)		
**Single-parent family**						0.17
	No	1410 (83.28)	229 (79.79)	1181 (84.00)		
	Yes	270 (15.95)	53 (18.47)	217 (15.43)		
	Missing data	13 (0.77)	5 (1.74)	8 (0.57)		
**Self-rated household financial situation relative to classmates**						<.001
	Good or very good	437 (25.81)	78 (27.18)	359 (25.53)		
	Average	1028 (60.72)	147 (51.22)	881 (62.66)		
	Poor or very poor	212 (12.52)	57 (19.86)	155 (11.02)		
	Missing data	16 (0.95)	5 (1.74)	11 (0.78)		
**Probable moderate or above depression^b^**						<.001
	No	1172 (69.23)	115 (40.07)	1056 (75.11)		
	Yes	521 (30.77)	172 (59.93)	349 (24.82)		
**Suicidal ideation**						<.001
	No	964 (56.94)	90 (31.36)	874 (62.16)		
	Yes	729 (43.06)	197 (68.64)	532 (37.84)		

^a^IGD: internet gaming disorder; IGD was defined as the endorsement of 5 or more out of the 9-item IGD checklist of the DSM-5 (Diagnostic and Statistical Manual of Mental Disorders [Fifth Edition]).

^b^Probable moderate or above depression was defined as the scale score of the Patient Health Questionnaire-9 score of ≥10.

### Prevalence of IGD, Probable Moderate or Above Depression, and Suicidal Ideation

The results are shown in Table 1. Over two-fifths (43.06%, 729/1693) reported suicidal ideation in the past year, and 16.95% (287/1693) were classified as IGD cases. Compared with their non-IGD counterparts (mean 13.46, SD 0.79), those with IGD (mean 13.60, SD 0.81) were more likely to be older (*P*=.01), belonging to the Chengdu sample (185/287, 64.46% vs 569/1406, 40.47%), and male (203/287, 70.73% vs 813/1406, 57.82%), and self-reported poor or very poor relative household financial situation (57/287, 19.86% vs 155/1406, 11.02%). The prevalence of probable moderate or above depression (PHQ-9 score≥10) was 30.77% (521/1693); the mean score of PHQ-9 was 7.29 (SD 6.82, range 0-27). IGD was significantly and positively associated with both probable moderate or above depression (172/287, 59.93% vs 349/1406, 24.82%; *P*<.001) and suicidal ideation (197/287, 68.64% vs 532/1406, 37.84%; *P*<.001).

### Levels of Psychosocial Resources and Psychosocial Problems

The mean scores of the resilience, social support, social anxiety, and loneliness scales were 23.07 (SD 8.73, range 0-40), 38.30 (SD 11.73, range 8-56), 12.70 (SD 7.14, range 0-27), and 15.78 (SD 5.04, range 8-32), respectively. The IGD group had significantly lower levels of psychosocial resources of resilience and social support and higher levels of psychosocial problems of social anxiety and loneliness than their counterparts (moderate to strong effect sizes; Cohen *d*=0.49-0.79; Table 2).

**Table 2:** Between-group differences in psychosocial resources and problems by IGD status (N=1693).

			Range		Overall, mean (SD)		IGD^a^, mean (SD)		Non-IGD, mean (SD)		*P* value		Cohen *d*
**Psychosocial resources**													
	Resilience	0-40		23.07 (8.74)		19.68 (7.84)		23.76 (8.75)		<.001		0.49	
	Social support	8-56		38.30 (11.73)		33.18 (11.63)		39.34 (11.48)		<.001		0.53	
**Psychosocial problems**													
	Social anxiety	0-27		12.70 (7.14)		15.80 (6.35)		12.08 (7.13)		<.001		0.55	
	Loneliness	8-32		15.78 (5.04)		18.85 (4.44)		15.16 (4.93)		<.001		0.79	

^a^IGD: internet gaming disorder.

### Correlations

Table 3 shows the significant positive correlations between resilience and social support (psychosocial coping resources; *r*=0.50) and between loneliness and social anxiety (psychosocial problems; *r*=0.48). The 2 psychosocial resource variables were negatively correlated with the 2 psychosocial problems (*r*=–0.40 to –0.13).

**Table 3:** Correlation analysis (n=1693).

			Resilience		Social support		Social anxiety
**Psychosocial resources**							
	Resilience	—^a^		—		—	
	Social support	0.50^b^		—		—	
**Psychosocial problems**							
	Social anxiety	−0.19^b^		−0.13^b^		—	
	Loneliness	−0.36^b^		−0.40^b^		0.48^b^	

^a^Not applicable.

^b^*P*<.001.

### Factors of Suicidal Ideation

Similar to the results of the univariate logistic regression analysis, multivariate logistic regression adjusting for the sociodemographics and probable moderate or above depression reported that increases in psychosocial coping resources of resilience (adjusted OR 0.97, 95% CI 0.96-0.98) and social support (adjusted OR 0.97, 95% CI 0.96-0.98) were associated with a lower risk of suicidal ideation while increases in IGD (adjusted OR 2.42, 95% CI 1.73-3.37) and psychosocial problems of social anxiety (adjusted OR 1.07, 95% CI 1.05-1.09) and loneliness (adjusted OR 1.13, 95% CI 1.10-1.16) were associated with a higher risk of suicidal ideation (Table 4).

**Table 4:** Univariate and multivariate logistic regression analysis on the individual associations between independent variables and suicidal ideation (n=1693). There are 10 individual models in total, 5 for univariate logistic regression and the other 5 for multivariate logistic regression.

		Unadjusted OR (95% CI)	*P* value	Adjusted OR^a^ (95% CI)	*P* value
**Psychosocial resources**					
	Resilience	0.95 (0.93-0.96)	<.001	0.97 (0.96-0.98)	<.001
	Social support	0.95 (0.94-0.96)	<.001	0.97 (0.96-0.98)	<.001
**Psychosocial problems**					
	Social anxiety	1.10 (1.09-1.12)	<.001	1.07 (1.05-1.09)	<.001
	Loneliness	1.21 (1.18-1.24)	<.001	1.13 (1.10-1.16)	<.001
**Internet gaming disorder**					
	No	Reference=1.0	—^b^	Reference=1.0	—
	Yes	3.60 (2.74-4.72)	<.001	2.42 (1.73-3.37)	<.001

^a^The adjusted models were adjusted for background factors (ie, age, studied city, sex, living arrangement, single-parent family, and household financial situation relative to classmates) and probable moderate or above depression (Patient Health Questionnaire-9 score≥10).

^b^Not applicable.

### Structural Equation Modeling

**Figure 1:**
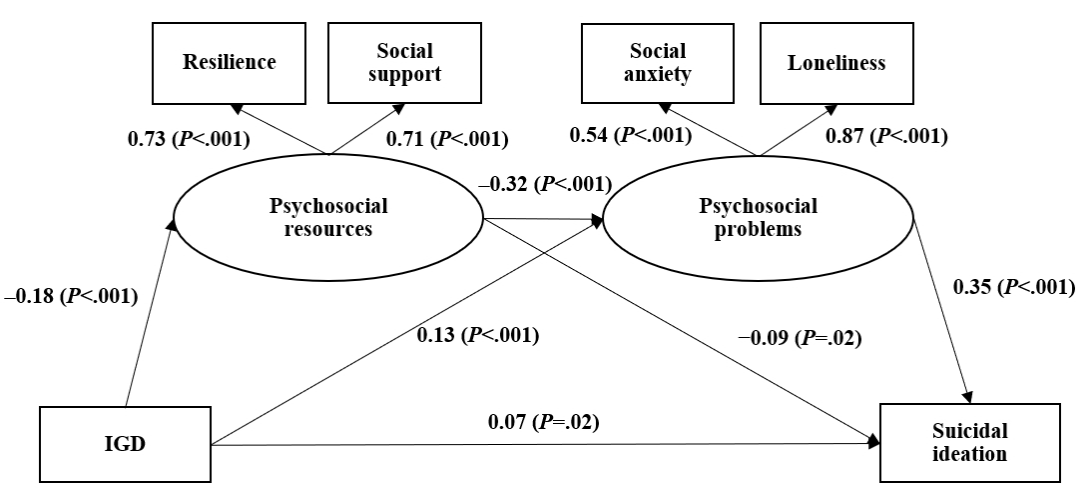
Structural equation modeling testing the mediation effect of psychosocial coping resources and psychosocial problems between internet gaming disorder (IGD) and suicidal ideation (standard coefficients were reported).

Figure 1 presents the results of SEM testing the proposed mediation effects of psychosocial coping resources and psychosocial problems between IGD and suicidal ideation. The model showed satisfactory goodness-of-fit (*χ*^2^_21_=176.21, *P*<.001; CFI=0.92; RMSEA=0.07), with factor loadings of the 2 latent variables ranging from 0.54 to 0.87 (all *P*<.001). The results showed that IGD had a significant direct effect on suicidal ideation (β=.07, *P*=.02). In addition, IGD was linked to suicidal ideation through 3 indirect paths: (1) a 2-step mediation path involving a negative association between IGD and the latent variable of psychosocial coping resources (β=–.18, *P*<.001), which was negatively associated with suicidal ideation (β=–.09, *P*=.02); (2) a 2-step mediation path involving a positive association between IGD and the latent variable of psychosocial problems (β=.13, *P*<.001), which was positively associated with suicidal ideation (β=.35, *P*<.001); and (3) a 3-step serial path involving a negative association between IGD and the latent variable of psychosocial coping resources (β=–.18, *P*<.001) and then a negative association between psychosocial coping resources and psychosocial problems (β=–.32, *P*<.001), which was in turn positively associated with suicidal ideation (β=.35, *P*<.001). Partial mediations were found as the direct path from IGD to suicidal ideation was of statistical significance. The corresponding mediation effect size of the above 3 indirect paths were 10.7% (indirect effect/total effect: 0.016/0.15), 30.0% (0.05/0.15), and 13.3% (β=.02/0.15, *P*<.001), respectively.

## Discussion

### Principal Findings

This survey was conducted among Chinese adolescents and found that IGD increased the risk of suicidal ideation. Both protective factors of psychosocial resources (resilience and social support) and risk factors of psychosocial problems (social anxiety and loneliness) for suicidal ideation were also identified. Furthermore, this study was novel to reveal the serial mediation mechanisms through psychosocial resources and psychosocial problems between IGD and suicidal ideation, which provide empirical evidence for future interventions targeting suicidal ideation as a negative consequence of IGD.

### Comparison With Previous Work

This study observed the alarmingly high prevalence of suicidal ideation of 43.06% (729/1693) among junior middle school students in 2 Chinese cities, which was higher than that of studies previously conducted in adolescents of other Chinese cities (18.2%-27.2%) [12,65], Poland (24.7%) [51], and Japan (25.7%) [66] based on the same measurement of suicidal ideation. Notably, this study was conducted before the COVID-19 pandemic period. A study conducted on Canadian adolescents reported that the prevalence of suicidal ideation has tripled to 44% since the beginning of the COVID-19 pandemic [67]. The prevalence of adolescent suicidal ideation during the pandemic period in China might have also increased due to the suspension of classes and interruptions of social interactions and activities. As suicidal ideation predicts suicidal behavior, early detection, together with timely and evidence-based secondary interventions, are greatly warranted [68,69].

Corroborating previous cross-sectional and longitudinal findings [11,13], this study found a strong association between adolescent IGD and suicidal ideation. In addition, the high prevalence of IGD of 16.95% (287/1693) was reported among adolescent gamers in this study. Previous studies found a similarly high prevalence among adolescents in China [12,70], which was higher than the global prevalence estimate of 3.1% [71]. It was suggested by some researchers that Asian adolescents tend to have higher IGD prevalence than those in Western countries [72]. Such findings highlight the importance of preventing and treating adolescent IGD, which was associated with various negative consequences, including depression, sleep problems, and physical violence [10,23]. A review summarized several efficacious intervention strategies for the purpose, including psychotherapies such as mindfulness, gaming-specific cognitive behavioral therapy, basic counseling and support groups, family therapy, gaming abstinence, self-discovery camp, and residential camp and parent management [73].

A novel finding of this study is about the mediation mechanisms between IGD and suicidal ideation through psychosocial coping resources (resilience and social support) and psychosocial problems (social anxiety and loneliness). As the direct effect of IGD on suicidal ideation remained statistically significant, the association between IGD and suicidal ideation was partially mediated by 3 indirect paths. These findings add empirical evidence to support the theoretical postulations of the Interpersonal-Psychological Theory of Suicide [24], the Resource Deterioration Model [30,31], and the Conservation of Resource Theory [32-35] aforementioned in the Introduction section. In comparison, the 2-step indirect path that IGD increased psychosocial problems, which then increased suicidal ideation, explained a larger proportion of the association between IGD and suicidal ideation than the other 2-step path and the 3-step path. Psychosocial problems might thus have explained more harmful effects of IGD on suicidal ideation than losses in psychosocial resources. Notably, as these 3 indirect paths only showed an overall partial mediation effect, other unstudied plausible mediators may exist. Examples include health problems (eg, sleep quality), psychological well-being (eg, life satisfaction and quality of life), and stress, which were associated with both IGD and determinants of suicidal ideation [74,75].

Notably, the multivariate logistic regression analyses and SEM in this study were adjusted for depression in addition to sociodemographics. As this study aimed to understand the relationship between IGD and suicidal ideation, given the known associations between depression and IGD [10], suicidal ideation [43], and the 4 psychosocial mediators [45,76], the adjustment for depression could ensure that the relationship of interest would not have been confounded by depression. A similar approach has been used in the extant literature investigating the independent associations between both risk and protective factors and suicidal ideation [43,77].

This study has added empirical evidence to the knowledge that psychosocial coping resources of resilience and social support are protective factors, while psychosocial problems are risk factors for adolescent suicidal ideation. There are thus reasons to believe that modification of these 4 psychosocial variables would reduce suicidal ideation directly and indirectly by reducing the harmful impact of IGD on suicidal ideation. Enhancement of psychosocial resources would also reduce psychosocial problems and the impact of IGD on psychosocial problems. Furthermore, according to a meta-analysis, interventions focusing on psychoeducation and social cognitions were efficacious in reducing loneliness and social isolation. Related interventions included guided social group participation, psychological therapy sessions, self-management, and training enhancing social and emotional skills, social interaction, and social support [78]. Another meta-analysis reported that resilience-improving interventions based on a combination of cognitive behavioral therapy and mindfulness techniques were able to improve resilience and overall well-being (eg, lower levels of anxiety) [79]. Future interventions may consider these approaches.

### Strengths and Limitations

This study was novel to investigate the harmful effect of IGD on suicidal ideation among Chinese adolescents and reveal the serial mediation mechanism potentially explaining this association through psychosocial resources and psychosocial problems. The results are implicative for future interventions. However, this study has other limitations. First, although using a single item to assess suicidal ideation has been widely used in the literature [67,68], such an assessment may be subject to misclassification (ie, false negative cases are those having suicidal ideation who were not screened out by the single-item question) in comparison with the multi-item assessment [68]. It may lead to an underestimation of the prevalence of suicidal ideation in this study and implies an alarmingly higher prevalence of suicidal ideation in Chinese adolescents. Second, social desirability bias may exist as the questions regarding IGD symptoms and suicidal ideation are sensitive and socially undesirable. Third, the cross-sectional study design precluded inference of temporal or causal relationships; future longitudinal and intervention studies are needed to confirm the findings. Fourth, the study population was conveniently selected from 7 schools in 2 Chinese cities; generalization of the results to other regions and populations in China and other countries should be made cautiously. Fifth, the relationships among IGD, psychosocial coping resources, and psychosocial problems may be bidirectional, as the literature also reported that resilience, social support, social anxiety, and loneliness were predictors of IGD [22,80-83]. Finally, some potential mediators between IGD and suicidal ideation (eg, sleep quality, psychological well-being, and stress) had not been investigated in this study.

### Conclusions

In conclusion, this study observed alarmingly high levels of both IGD and suicidal ideation among adolescents in 2 Chinese metropolises. Very importantly and corroborating previous studies, a strong and positive association between IGD and suicidal ideation was found in this study, indicating that IGD may potentially lead to adolescent suicide. Adolescents and stakeholders (parents, teachers, and health workers) need to be made aware of the potential risk of IGD in elevating suicidal ideation. Future studies should also investigate factors moderating such a relationship. Furthermore, it is important and novel that this study revealed the mechanism of such an association, which included 3 indirect paths through psychosocial resources (resilience and social support) and psychosocial problems (social anxiety and loneliness) between IGD and suicidal ideation. Longitudinal and intervention studies are warranted to confirm these findings and explore other mechanisms. Tailor-made modifications of these psychosocial variables (especially psychosocial problems) may directly and indirectly reduce the harmful impacts of IGD on suicidal ideation.

## Abbreviations

CFI: comparative fit index
DSM-5: Diagnostic and Statistical Manual of Mental Disorders (Fifth Edition)
ICD-11: International Classification of Diseases (11th Edition)
IGD: internet gaming disorder
RMSEA: root-mean-square error of approximation
SEM: structural equation modeling
UCLA: University of California, Los Angeles

## Data Availability

The data sets generated during and/or analyzed during this study are available from the corresponding author on reasonable request.
